# The circular RNA circSPARC enhances the migration and proliferation of colorectal cancer by regulating the JAK/STAT pathway

**DOI:** 10.1186/s12943-021-01375-x

**Published:** 2021-06-01

**Authors:** Jiaqi Wang, Yi Zhang, Hu Song, Hang Yin, Tao Jiang, Yixin Xu, Lianyu Liu, Hongyu Wang, Hong Gao, Renhao Wang, Jun Song

**Affiliations:** 1 Department of General Surgery, The Affiliated Hospital of Xuzhou Medical University, Xuzhou, 221002 Jiangsu Province China; 2 Institute of Digestive Diseases, Xuzhou Medical University, Xuzhou, 221002 Jiangsu Province China

**Keywords:** Colorectal cancer, circRNA, circSPARC, JAK/STAT signalling pathway, Biomarker

## Abstract

**Background:**

Noncoding RNAs such as circular RNAs (circRNAs) are abundant in the human body and influence the occurrence and development of various diseases. However, the biological functions of circRNAs in colorectal cancer (CRC) are largely unknown.

**Methods:**

RT-qPCR was used to detect the expression of circRNAs and mRNA in CRC cells and tissues. Fluorescence in situ hybridization (FISH) was used to analyze the location of circSPARC. Function-based experiments were performed using circSPARC knockdown and overexpression cell lines in vitro and in vivo, including CCK8, colony formation, transwell and metastasis models. Mechanistically, luciferase reporter assay, western blots, RNA immunoprecipitation (RIP), Chromatin isolation by RNA purification (ChIRP) and immunohistochemical stainings were performed.

**Results:**

CircSPARC was upregulated in both the tissues and plasma of CRC patients. High expression of circSPARC was associated with advanced TNM stage, lymph node metastases, and poor survival. Silencing circSPARC inhibited CRC cell migration and proliferation in vitro and vivo. Mechanistically, circSPARC sponged miR-485-3p to upregulate JAK2 expression and ultimately contribute to the accumulation of phosphorylated (p)-STAT3. Besides, circSPARC recruited FUS, which facilitated the nuclear translocation of p-STAT3.

**Conclusions:**

These findings suggest that circSPARC might serve as a potential diagnostic and prognostic biomarker and a therapeutic target for CRC treatment by regulating JAK2/STAT3 pathway.

**Supplementary Information:**

The online version contains supplementary material available at 10.1186/s12943-021-01375-x.

## Background

Colorectal cancer (CRC) is one of the most common malignant cancers with high incidence and mortality worldwide [1]. In addition to heredity, obesity, physical inactivity, poor diet, alcohol consumption and smoking are all factors responsible for CRC development. Moreover, the rising incidence of CRC has tended to shift to developing countries and younger age groups [2]. Currently, with the development of technologies, such as cancer biomarkers, colonoscopy, laparoscopic surgery and chemoradiotherapy [3], the treatment options for CRC patients have increased. However, post-treatment problems including recurrence, metastasis and chemoresistance are associated with poor prognosis and need to be solved [4]. It has been reported that the progression of CRC is closely related to the dysregulation of genes and signalling pathways [5]. Thus, to achieve early diagnosis and better prognosis, novel biomarkers and therapeutic targets of CRC should be investigated.

Circular RNAs (circRNAs), which were first identified more than four decades ago [6], are a class of endogenous noncoding RNAs (ncRNAs) with a closed-loop structure that lack 5′ end caps and 3′ end poly(A) tails [7]. A recent study stated that circRNAs may not have a simple circular structure but may contain double-stranded internal complementary base-pairing sequences (ICBPS), which was explored in many circRNAs [8]. Due to their unique structure, circRNAs are more stable and more resistant to degradation than mRNAs, and cannot be amplified in genomic DNA [9]. The development of high-throughput RNA sequencing and the application of bioinformatics technology have resulted in the confirmation of circRNAs as important molecules that can be abnormally expressed and associated with poor prognosis and chemoresistance in cancers [10]. According to recent research, circRNAs mainly function as microRNA (miRNA) sponges through the presence of miRNA response elements (MREs), specific sequence motifs complementary to particular miRNAs, and act as competing endogenous RNAs (ceRNAs) to alleviate the miRNA-mediated repression of downstream target mRNAs [11]. Similar to their role as miRNA sponges, some circRNAs have binding sites for RNA-binding proteins (RBPs) and can recruit or repress RBPs. In addition, a few studies have discovered that some circRNAs have transcriptional and post-transcriptional regulatory functions, while others can even be translated into proteins [12].

The Janus kinase (JAK)/signal transducer and activator of transcription (STAT) signalling pathway is involved in cancer cell migration, growth and differentiation [13]. When activated by cytokines, JAKs in turn activate signalling molecules such as STATs. Activated STATs translocate from the cytoplasm to the nucleus, where they enhance the transcription of target genes. The most important molecules in this signalling pathway are JAK2 and STAT3. Upon activation by cytokines, several JAK2 molecules are brought in close proximity and are autophosphorylated to form phosphorylated (p)-JAK2. Then, p-JAK2 activates STAT3 by phosphorylation, and the translocation of p-STAT3 into the nucleus results in the enhanced transcription of several genes, such as epithelial-mesenchymal transition (EMT)-related genes, c-myc, MMP2, and p53. In CRC, the JAK/STAT signalling pathway can enhance cancer cell invasion, migration, growth, and chemoresistance and regulate cell progression, apoptosis and cell cycle progression.

In this study, RNA-seq was performed to analyse the expression of circRNAs in CRC tissues. The expression level of hsa_circ_0004104 was markedly upregulated in CRC tissues and was strongly correlated with the prognosis of CRC patients. Moreover, upregulation of hsa_circ_0004104 promoted cell proliferation and migration. Hsa_circ_0004104 was shown to sponge miR-485-3p, which can degrade JAK2 mRNA. Additionally, hsa_circ_0004104 could bind to FUS, an RBP that was identified to facilitate the translocation of p-STAT3 into the nucleus. In summary, hsa_circ_0004104 regulated the JAK2/STAT3 pathway by acting as a ceRNA to bind to miR-485-3p and FUS. Hsa_circ_0004104 could serve as a new biomarker for CRC diagnosis and prognosis and may be a potential therapeutic target for CRC patients.

## Materials and methods

### Clinical specimens

Between January 2012 and October 2014, 84 pairs of CRC tumour tissues and corresponding adjacent normal tissues and 40 preoperative and postoperative plasma samples were collected from patients with CRC who underwent surgery at Xuzhou Medical University Affiliated Hospital (Xuzhou, China). Seventeen pairs of colorectal adenoma tissues and corresponding adjacent normal tissues were collected at the same time. The patients did not receive any radiotherapy or chemotherapy before surgery. The samples were pathologically confirmed and quickly placed into liquid nitrogen after surgery until use. CRC patients were staged according to the TNM staging system (7th edition) of the American Joint Committee on Cancer. Details of the clinicopathological data are shown in Table 1. According to the sex- and age-matched criteria, fresh normal plasma samples were obtained from 40 healthy volunteers at Xuzhou Medical University Affiliated Hospital. When the expression of circSPARC was greater than the average, circSPARC was considered to be high, and circSPARC expression less than the average was considered to be low. The study protocol was approved by the Research Ethics Committee of Xuzhou Medical University. Clinical specimens were obtained with the informed consent of patients.

**Table 1:** The clinic-pathological factors of CRC patients

Characteristics	Number of cases	circSPARC expression		*P* value
		Low(*n* = 42)	High(*n* = 42)	
Gender				0.6611
Male	46	22	24	
Female	38	20	18	
Age (years)				0.3739
≤ 60	50	23	27	
>60	34	19	15	
Tumor size (cm)				**0.0045**
≤ 5	45	29	16	
>5	39	13	26	
Tumor invasion				**0.0085**
T1-T2	38	25	13	
T3-T4	46	17	29	
Lymphatic metastasis				**0.0140**
Negative	33	22	11	
Positive	51	20	31	
Distant metastasis				**0.0484**
M0	77	41	36	
M1	7	1	6	
Clinical stage				**0.0149**
I-II	35	23	12	
III-IV	49	19	30	

### Cell lines and culture

Human CRC cell lines (HCT116, SW620, SW480, DLD1, HT-29, and LoVo) were purchased from the Cell Bank of the Chinese Academy of Science (Shanghai, China). The human normal colorectal epithelial cell line FHC was obtained from the American Type Culture Collection (Manassas, VA, USA). Cell lines were cultured in the appropriate medium supplemented with 10% foetal bovine serum (FBS; Gibco, NY, USA) and 1% antibiotic/antimycotic solution and maintained in an incubator at 37 °C with 5% CO2 in a humidified atmosphere. DLD1, LoVo and HT-29 cells were maintained in RPMI-1640 medium (Gibco), FHC and SW480 cells were cultured in Dulbecco’s modified Eagle medium (DMEM; Gibco), HCT116 cells were maintained in McCoy’s 5A medium (KeyGEN, Nanjing, China) and SW620 cells were maintained in L-15 medium (KeyGEN).

### Cell transfection

Negative control siRNA (si-NC), two different circSPARC siRNAs (si-circSPARC #1 and #2), FUS siRNA, miR-485-3p mimics and miR-485-3p inhibitor were purchased from Gene Pharma (Shanghai, China). Cells were transfected with siRNA at 30–50% confluence using siLentFect Lipid Reagent (Bio-Rad, CA, USA). CircSPARC and FUS were amplified from human cDNA template and cloned into the pcDNA3.1(+) vector (Invitrogen, USA). HCT116 and DLD1 cells were grown to 90% confluence before being transiently transfected with plasmids using Lipofectamine 2000 (Invitrogen) according to the manufacturer’s protocol. At 24–48 h post transfection, the cells were harvested for subsequent analysis.

### Cell invasion, migration, wound healing, proliferation and colony formation assays

The cell migration and invasion abilities were evaluated by a modified bicameral culture system with a pore size of 8 μm. Transwell inserts (Corning Incorporated, USA) with or without Matrigel (BD Biosciences, USA) coating were used to perform the invasion or migration assays, respectively. The transfected cells were seeded into the Transwell inserts with serum-free medium and 10% FBS medium put at the bottom. After 24 h culture, the cells were fixed with 4% paraformaldehyde solution and stained with 0.1% crystal violet. Then, we used an Olympus microscope to obtain images at a magnification of 100× and used ImageJ software to calculate the number of cells penetrating the pores. All experiments were carried out three times.

For wound healing assay, 100% density of cells were cultured in six-well plates with serum-free medium, and cell monolayer was subsequently scratched with a 200ul pipette tip. Representative images of cell migration were captured at 0 h, 12 h and 24 h after injury. Remodeling was measured as the diminishing distance across the induced injury area normalized to the 0 h control and expressed as a relative migration rate. For each one, the experiments were repeated for at least three times with three replicates.

Cell proliferation was assessed with the Cell Counting Kit-8 (CCK-8) assay (APExBIO). Equal numbers of cells were seeded into 96-well plates. CCK-8 solution (10 μl per well) with 100 μl serum-free medium was added every 24 h, and the plates were incubated at 37 °C for 2 h. The optical density (OD) was then measured at 450 nm. For the colony formation assay, cells transfected similarly were plated in each plate and cultured in the appropriate medium containing 10% FBS for approximately 14 days, and the medium was replaced every 5 days. After 14 days, the colonies were fixed with methanol and stained with 0.1% crystal violet (Vicmed, China). The colony formation rate was determined by counting the number of stained colonies.

### Quantitative real-time PCR (RT-qPCR) and RT-PCR

According to the manufacturer’s instructions, total RNA from tissues, plasma and cells was isolated using TRIzol reagent (TaKaRa, China) and quantified. CircRNAs, miRNAs and mRNAs were reverse transcribed by using PrimeScript RT Master Mix (TaKaRa) in accordance with the manufacturer’s protocol. The relative quantification of circRNAs was carried out by the 2^-ΔΔCT^ method, and 18S rRNA was used as an internal control. The expression levels of miRNAs were normalized to the levels of the internal control U6 by the 2^-ΔΔCT^ method. The expression levels of related mRNAs were normalized to the levels of the internal control GAPDH by the 2^-ΔΔCT^ method. The reactions were performed independently in triplicate. Quantitative PCR assays were carried out on ABI StepOne (Carlsbad, CA, USA). The divergent, convergent and 18S primers were amplified in both cDNA and genomic DNA. The products were detected by AGE (agarose gel electrophoresis). All the primer sequences are listed in Additional file (Table S1).

### Western blotting, coimmunoprecipitation (co-IP) and antibodies

Cells were harvested with RIPA lysis buffer (Beyotime, China) supplemented with PMSF, phosphatase inhibitor cocktail and protease inhibitor cocktail (Sigma-Aldrich, MO, USA). The lysates were cleared by centrifugation at 13,000×g for 15 min at 4 °C. The cell protein lysates were quantified, separated by SDS-polyacrylamide gel electrophoresis, transferred to a nitrocellulose filter membrane, blocked with 5% skim milk (BD Biosciences) in Tris-buffered saline with 0.05% Tween-20, and probed with specific antibodies. The signals were detected using Chemistar™ High-sig ECL Western Blot Substrate (Tanon, Shanghai, China). Similarly, human tissues were ground and then prepared in RIPA buffer as mentioned above. For co-IP, cell lysates (1000 μg) containing a cocktail of protease/phosphatase inhibitors were rotated overnight for IP with anti-FUS IgG, anti-STAT3 IgG and rabbit IgG (Beyotime, China). Then, 30 μl of protein A/G agarose beads (Santa Cruz Biotechnology, USA) was added to the cell lysates and incubated for 4 h at 4 °C. The beads were washed with lysis buffer three times. The IP complexes were analysed by western blotting. All the antibodies used against the proteins are shown in Additional file (Table S2).

### RNA-fluorescence in situ hybridization (RNA FISH)

HCT116 cells were washed twice with phosphate-buffered saline (PBS) and fixed in 4% formaldehyde for 15 min. The fixed cells were permeabilized with Triton X-100 and dehydrated by an ascending series of ethanol concentrations. The cells were then incubated with 50 nmol probe, which was labelled with Cy3 at the 5′ end in hybridization buffer at 73 °C for 5 min. The cells were hybridized at 37 °C for 14 h, washed and dehydrated. After adding DAPI working solution, the cells were scanned and imaged. RNA FISH probes were designed and synthesized by Sangon Biotech (Shanghai, China). The probe sequences are listed in Table S1.

### RNA immunoprecipitation (RIP) assay

The RIP experiments were conducted by using a Magna RNA-binding protein immunoprecipitation kit (Millipore, Billerica, MA, USA) following the manufacturer’s instructions. In brief, cell extracts were mixed with protein A/G beads conjugated to an antibody against FUS or IgG (negative control). Then, the precipitated RNAs were analysed by qPCR.

### Dual luciferase reporter assay

A dual luciferase reporter assay was used to evaluate the direct binding between circSPARC and miRNAs as well as JAK2 and miRNAs. The circSPARC sequence was cloned into the psiCHECK-2 (GenePharma, China) vector, which contains the firefly luciferase gene (hLuc+) and Renilla luciferase gene (hRluc).. The NC vector or circSPARC vector was co-transfected with each miRNA mimic. The relative values of hLuc+ and hRluc were detected by Centro LB960 XS3 (Berthold, Germany). A luciferase reporter assay was used to determine whether JAK2 is the direct target of miR-485-3p. The 3’UTR sequence of JAK2 was cloned into the pcDNA3.0 vector. Next, the miR-485-3p mimic or NC was co-transfected with wild-type vector or the mutant vector. The relative luciferase value was also detected by Centro LB960 XS3 (Berthold, Germany).

### Chromatin isolation by RNA purification (ChIRP)

The ChIRP assay was performed using the Chromatin isolation by RNA purification (ChIRP) Kit (BersinBio™, China) following the manufacturer’s guidelines. Briefly, a total of 1 × 10^7^ cells were lysed in complete lysis buffer for each reaction, and the DNA was then sheared into small fragments through sonication. Then, the lysate was incubated with biotin-labelled probes that could hybridize with SATB2-AS1. We divided the probes into the ‘odd’ group and the ‘even’ group according to their order, and probes targeting LacZ were selected as nonspecific controls. Finally, the probes were extracted by streptavidin-coated magnetic beads, and the bound DNA was isolated for RT-qPCR.

### Chromatin immunoprecipitation (ChIP) assay

ChIP assay was performed according to the protocol of ChIP assay kit (Upstate Biotechnology, Lake Placid, NY). HCT116 cells cultured in 100 mm dish (about 1 × 107) were cross-linked by adding formaldehyde to final concentration of 1% and incubated in room temperature for 10 min, washed twice with cold PBS containing protease inhibitors, lysed by ChIP lysis buffer, sonicated to shear DNA at 4 °C to reduce the average length. Sonicated lysates were then diluted 10-fold with ChIP dilution buffer and reduced the non-specific binding with protein A agarose for 1 h at 4 °C, in this step, 20 μl of lysate were taken out as input control, then followed by incubation and with anti-CTCF or anti-IgG (as negative control) at 4 °C overnight with rotation. After reversal washes with a series of buffers, qRT-PCR was performed to amplify the 9 genomic regions of the SPARC flanking the potential CTCF binding sites.

### Immunofluorescence (IF) analysis

PBS-rinsed cells were fixed with 4% paraformaldehyde and then permeabilized using 0.25% Triton X-100 in PBS, followed by blocking with 1% BSA. After being washed three times with PBS, the cells were incubated with primary antibodies at 4 °C overnight. Subsequently, the cells were incubated with Cy3-conjugated secondary antibody for 1 h, and the nuclei were stained with DAPI. Images were captured under an immunofluorescence microscope (Olympus), with the analysis conducted by a Nikon and spot image acquisition system.

### Haematoxylin and eosin (H&E) and immunohistochemical staining

Paraffin-embedded lung tissues were sectioned at 5 μm thickness and then stained with H&E. Pulmonary tissues were cut into 4 μm sections, deparaffinized and rehydrated. For antigen retrieval, the slides were heated at 95 °C in 0.01 M citrate buffer (pH = 6.0), and 3% hydrogen peroxide was used to quench the peroxidase activity for 20 min. The sections were treated with normal goat serum, followed by incubation overnight with antibodies at 4 °C. After being rinsed with PBS, the sections were incubated with goat anti-rabbit IgG for 1 h and stained with 3,3′-diaminobenzidine (DAB; Zhongshan Biotech, Beijing, China). After haematoxylin counterstaining was completed, all sections were dehydrated and sealed. We used PBS instead of a primary antibody as a negative control. All images were recorded by an Olympus BX-51 light microscope.

### Lentiviral transduction and tumour xenograft experiments

Cells labelled with luciferase were infected with lentiviruses expressing GFP and carrying sh-NC or sh-circSPARC in the presence of 8 mg/ml polybrene (Gene Pharma). All animal experiments were approved by the Committee on the Ethics of Animal Experiments of Xuzhou Medical University. All animal studies complied with the National Institutes of Health Guide for the Care and Use of Laboratory Animals. Four- to six-week-old BALB/c female nude mice were provided by HFK Bioscience (Beijing, China). The animals in the experimental groups are divided randomly. In the subcutaneous tumour xenograft experiment, a total of 5 × 10^6^ sh-NC HCT116 cells and sh-circSPARC HCT116 cells with 200 μl Matrigel were injected into the same mouse. In the metastasis assay, sh-NC HCT116 cells and sh-circSPARC HCT116 cells suspended in 150 μl PBS were injected into mice through the tail vein. Tumour growth was measured every week. Mice in the subcutaneous injection groups were sacrificed after 1 month, and mice in the tail vein injection groups were sacrificed after 2 months.

### Statistical analysis

Statistical analysis was performed by SPSS 17.0 software (SPSS, USA), and images were acquired with GraphPad Prism 7 software (La Jolla, USA). The significance of the differences between the groups was evaluated by a paired two-tailed Student’s t-test or χ2 test. The correlation analysis between circSPARC and miR-485-3p was evaluated by Spearman’s test. The Kaplan-Meier method and log-rank test were used to evaluate overall survival (OS). Data represent the mean ± standard deviation (SD). Differences were considered statistically significant when *P* < 0.05 (* *P* < 0.05, ** *P* < 0.01, *** *P* < 0.001, and **** *P* < 0.0001).

## Results

### CircSPARC is overexpressed in CRC tissue and plasma

**Fig. 1:**
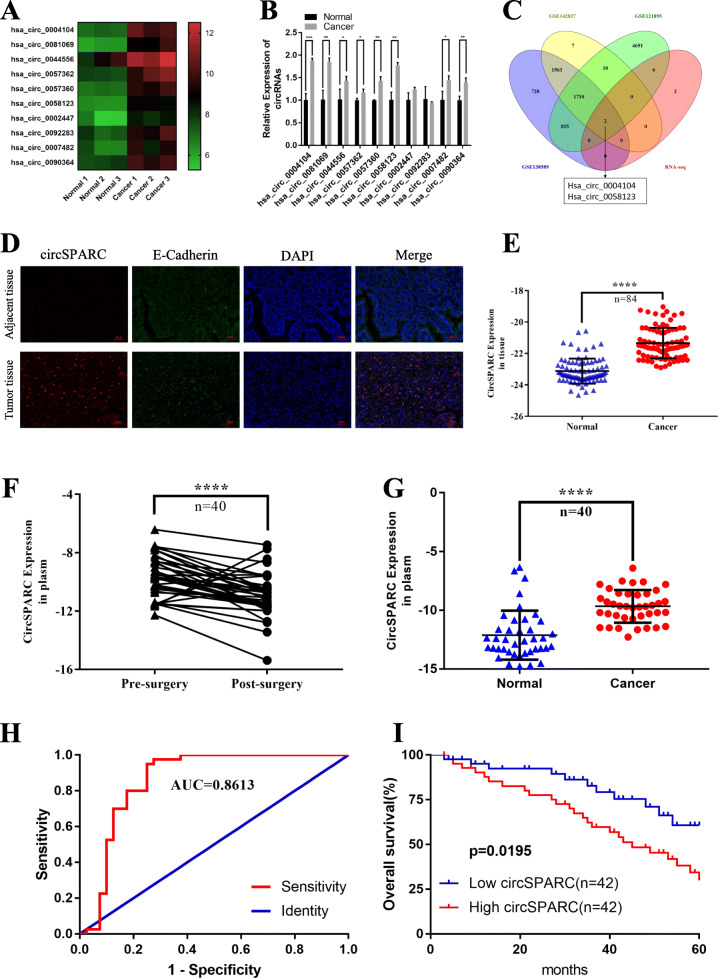
circSPARC was upregulated in CRC. **A.** Heatmap of RNA-Seq analysis of top 10 differentially high expressed circRNAs generated from three paired of CRC and corresponding adjacent non-tumoral tissues. Red in the heatmap denotes upregulation, green denotes downregulation. **B.** The expression of the top 10 differentially high expressed circRNAs were verified in CRC and corresponding adjacent non-tumoral tissues by RT-qPCR. **C.** The intersection of RNA-seq results and three data from the GEO database. **D.** The co-localization of epithelial marker E-Cadherin and circSPARC was applied by FISH-IF staining assay in CRC and corresponding adjacent non-tumoral tissues (200X). The results showed that circSPARC expressed higher in tumor tissues. **E.** RT-qPCR was utilized to analyze the circSPARC expression in 84 pairs of CRC tissues and corresponding adjacent non-tumoral tissues. **F.** Expression of circSPARC was detected by RT-qPCR in 40 pairs of pre-surgery and post-surgery patients plasm. **G.** RT-qPCR was applied to analyze the circSPARC expression in 40 pairs of CRC patients and general people plasm. **H.** ROC analysis of the prognostic sensitivity and specificity for CRC patients and general people detected by the expression of circSPARC in plasm. **I.** Kaplan-Meier overall survival curves according to circSPARC expression in our cohort. *P < 0.05, **P < 0.01, ***P < 0.001 and ****P < 0.0001

To investigate the differential expression of circRNAs in CRC, we studied the RNA sequencing data of three pairs of human CRC tissues and paired normal colorectal tissues. We identified the upregulated circRNAs with an average normal tissue read count of more than 100 and then sorted them by fold change. Then, we filtered the 10 most upregulated circRNAs (Fig. 1A) and verified them by RT-qPCR in one pair of CRC tissues and matched adjacent normal tissues (Fig. 1B). Furthermore, we analysed online datasets of circRNA profiles in CRC tissues (GSE121895, GSE138589 and GSE142837). We found that two circRNAs (hsa_circ_0004104 and hsa_circ_0058123), which were overexpressed in CRC tissues compared to peritumour tissues, were included both in the datasets and the RNA-seq results (Fig. 1C). Among them, hsa_circ_0004104 (termed circSPARC) was predicted to be the most upregulated circRNA according to the above results. Then, the levels of circSPARC in 84 paired CRC tissues and adjacent normal tissues, 17 paired colorectal adenoma tissues and adjacent normal tissues, 40 paired plasma samples from the general population and CRC patients and 40 paired CRC patient plasma samples before and after surgery were detected. FISH-IF staining assays in tissue, which co-localized of epithelial marker E-Cadherin with the circSPARC, showed that circSPARC was expressed at a high level in tumour tissue (Fig. 1D). The results verified that circSPARC was significantly upregulated in tumour tissues and patient preoperative plasma while it was slightly upregulated in adenoma tissues (Fig. 1E-G, Additional file Fig. S1A). This indicates that circSPARC may promote the progression of CRC. Furthermore, the ROC curve showed that circSPARC had potential clinical significance as a tumour marker and could be used in diagnosis and to monitor therapeutic efficacy (Fig. 1H).

Further analysis of clinical data revealed a correlation between the expression of circSPARC and the clinicopathological characteristics of CRC patients. Correlation analysis demonstrated that high levels of circSPARC were associated with tumour size (*P* < 0.005), tumour invasion (*P* < 0.001), lymphatic metastasis (*P* < 0.05), distant metastasis (*P* < 0.05) and Clinical stage (*P* < 0.05). However, there was no significant relationship between circSPARC expression and other factors, including age (*P* = 0.3739) and sex (*P* = 0.6611) (Table 1). Kaplan-Meier analysis was performed to further determine whether the increase in circSPARC staining in patients with CRC was associated with poor prognosis, which showed that high levels of circSPARC in our cohort were associated with decreased OS (Fig. 1I). Therefore, these results showed that circSPARC may play an important role in the progression and homeostasis of CRC.

### Characterization of circSPARC in CRC

**Fig. 2:**
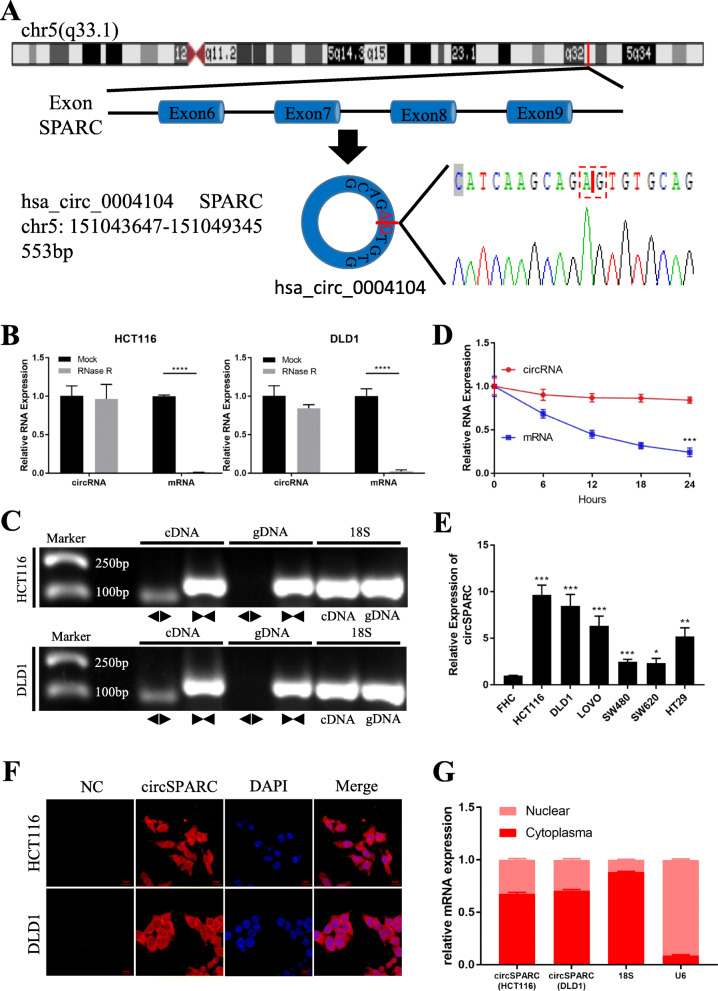
The characteristics of circSPARC. **A.** Schematic illustration indicating the generation of circSPARC from its host gene, and validation by Sanger sequencing. **B.** RT-qPCR for the abundance of circSPARC and its host gene mRNA in CRC cells treated with RNase R. **C.** PCR assay with divergent (◀▶) and convergent (▶◀) primers showing the amplification of circSPARC from cDNA or gDNA of CRC cell lines, while 18 s was used as a positive control. It can be observed that circSPARC was amplified in cDNA but not in gDNA while linear SPARC was amplified in both. **D.** Transcript half-life of circSPARC and its linear transcript in HCT116 cells treated with the transcription inhibitor actinomycin D. **E.** RT-qPCR indicated the circSPARC expressed higher in CRC cell lines (HCT116, DLD1, LoVo, SW480, SW620 and HT29) than in the normal colorectal epithelium cell line (FHC). **F** and **G.** FISH assay and nuclear-cytoplasm separation RT-qPCR data showed that circSPARC existed in both cytoplasm and nucleus, but primarily localized in the cytoplasm in CRC cells. *P < 0.05, **P < 0.01, ***P < 0.001 and ****P < 0.0001

Hsa_circ_0004104 is generated from exons 6–9 of the SPARC gene located on chr5:151,043,647-151,049,345 and is 553 bp in length (Fig. 2A). Since its unique circular structure, circRNA is more resistant to RNase R than their parental linear genes [14]. Endogenous circSPARC was resistant to RNase R digestion, while the amount of the linear mRNA of SPARC in CRC cells (HCT116 and DLD1) was significantly reduced by RNase R treatment (Fig. 2B). Another characteristic of circRNA is that it can be detected in cDNA only but not in genomic DNA [15]. CircSPARC was validated by the detection of PCR amplification products using divergent primers from cDNA, but not from genomic DNA, of CRC cell lines, and 18S was used as positive control (Fig. 2C). RT-qPCR indicated that the transcript half-life of circSPARC was longer than that of the linear transcript (Fig. 2D). We further compared circSPARC expression between CRC cells and normal human colorectal epithelial cells (Fig. 2E). CircSPARC was dramatically higher in all tested CRC cells (HCT116, DLD1, LoVo, SW480, SW620, and HT29) than in normal human colorectal epithelial FHC cells, and HCT116, DLD1, and LoVo cells had the highest expression of this circRNA. Furthermore, we determined that circSPARC was primarily located in the cytoplasm through FISH experiments and nuclear mass separation assays in HCT116 and DLD1 cells (Fig. 2F, G). Taken together, these results indicate that circSPARC is truly a circRNA and is mainly located in the cytoplasm.

### CircSPARC promotes the invasion and proliferation of CRC cells in vitro

**Fig. 3:**
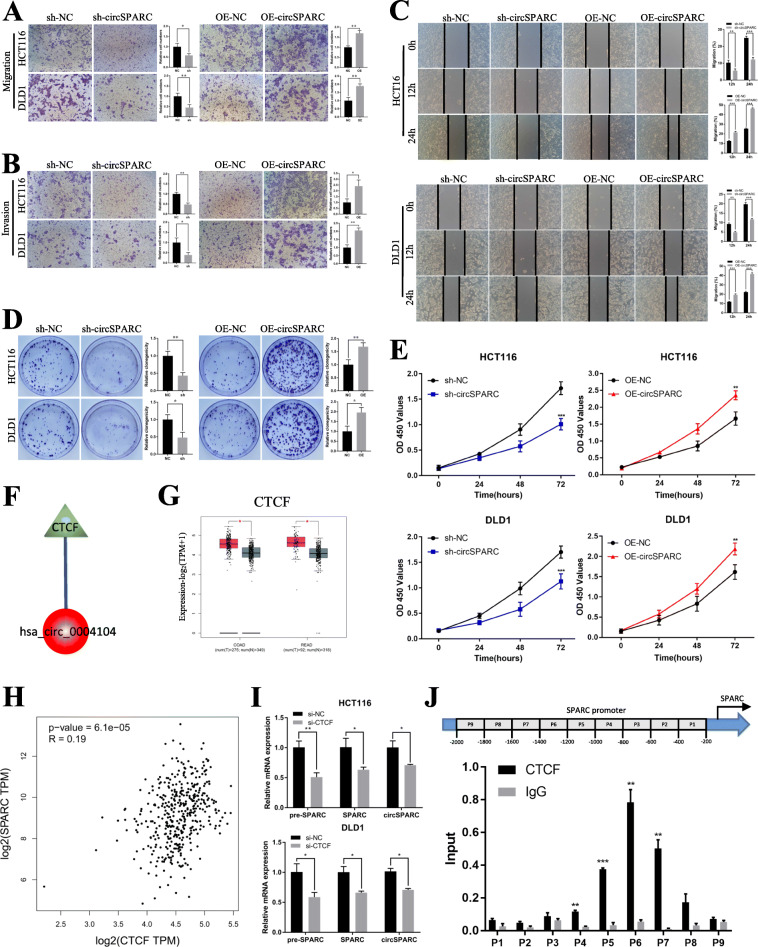
circSPARC regulated the proliferation and invasion of CRC cells and CTCF may function as the upstream of circSPARC. **A** and **B.** The transwell assay was performed for invasion and migration of CRC cells. **C.** The wound healing assay was performed for migration of CRC cells. **D.** The colony formation assay was performed for proliferation of CRC cells. **E.** The CCK-8 assay was performed for proliferation of CRC cells. **F.** TRcirc tool predicted that CTCF can function as the potential upstream of circSPARC. **G.** GEPIA database exhibited that CTCF is upregulated in CRC. **H.** GEPIA database showed that CTCF has a positive correlation with SPARC in CRC. **I.** RT-qPCR results showed that the level of SPARC pre-mRNA, circSPARC and SPARC mRNA were all decreased when CTCF was knockdown in HCT116 and DLD1 cells. **J.** P1-P9 showed the regions of SPARC promoter detected by the paired primers. ChIP assay revealed that CTCF mainly binds at P5, P6 and P7. *P < 0.05, **P < 0.01, ***P < 0.001 and ****P < 0.0001

To determine the biological roles of circSPARC in CRC cells, we first designed two siRNAs (si-circSPARC#1 and si-circSPARC#2) and one overexpression plasmid (OE-circSPARC) targeting circSPARC and transfected them into the CRC cell lines (HCT116 and DLD1) with the highest circSPARC expression level to modify circSPARC expression. The RT-qPCR results showed that transfection of si-circSPARC#1 or OE-circSPARC resulted in statistically significant changes in circSPARC expression levels, while the effect of si-circSPARC#2 seemed to be weaker. Then, we successfully established a stable interference system for circSPARC by lentiviral transduction (sh-circSPARC) in HCT116 and DLD1 cells. Meanwhile, the parental gene SPARC was detected at the same time and the level of endogenous SPARC mRNA and protein expressed stably when the expression of circSPARC was changed. All the results above were showed in Additional file Fig. S1B-D. In order to exclude the influence of proliferation on the ability of cell invasion and migration, the cells were first cultured in serum-free medium for 24 h and cell activity was detected at 0 h, 12 h and 24 h. The CCK-8 assay results showed that compared with the control group, cell proliferation rates were not statistically significant within 24 h after knockdown or overexpression of circSPARC (Additional file Fig. S1E). By performing Transwell and Wound healing assays, we demonstrated that interference with circSPARC significantly inhibited the migration and invasion abilities of CRC cells, whereas overexpression of circSPARC promoted the migration and invasion abilities of CRC cells (Fig. 3A-C). Furthermore, the CCK-8 assay was used to detect the viability of HCT116 and DLD1 cells at 0, 24, 48 and 72 h, and our results showed that silencing circSPARC significantly inhibited the viability (OD value) of HCT116 and DLD1 cells at 450 nm; the clone formation assay indicated that silencing circSPARC significantly inhibited the clone formation ability. In contrast, circSPARC overexpression increased the viability and clone formation ability of CRC cells (Fig. 3D, E).

### CircSPARC is transcriptionally regulated by CTCF

Considering that circSPARC was upregulated in CRC, we predicted the upstream regulator of circSPARC in CRC. The TRcirc (https://www.licpathway.net/TRCirc) exhibited that CCCTC binding factor (CTCF) was the only transcription factor (TF) modulating circSPARC transcription (Fig. 3F). Recently, CTCF was proved as an oncogene in CRC [16]. Here, Gene Expression Profiling Interactive Analysis (GEPIA, https://gepia.cancer-pku.cn/), was used to explore the expression of SPARC, the parental gene of circSPARC, and CTCF in The Cancer Genome Atlas (TCGA) database. Results indicated that both CTCF and SPARC were upregulated in CRC (Fig. 3G, Additional file Fig. S2A) and there was a positive correlation between SPARC and CTCF expression predicted by GEPIA and Starbase (https://starbase.sysu.edu.cn/) (Fig. 3H, Additional file Fig. S2B). Next, we verified the expression of CTCF in CRC cells and designed the siRNA of CTCF. It showed that CTCF was overexpressed in CRC cells compared with FHC cells (Additional file Fig. S2C), and the effect of si-CTCF showed in Additional file Fig. S2D. Furthermore, the results of RT-qPCR were observed that when CTCF was knockdown, the level of SPARC pre-mRNA, SPARC mRNA and circSPARC decreased all (Fig. 3I). It might indicate that as a TF, CTCF influenced the transcription progression of SPARC pre-mRNA, which result in the changes of downstream SPARC mRNA and circSPARC. Based on this hypothesis, ChIP assay was carried out to investigate the fragment of SPARC promoter that can be bonded together with CTCF. As shown in Fig. 3J, nine fragments were designed as the potential binding regions in SPARC promoter and CTCF was mainly bound to the P5-P7 binding regions of the SPARC promoter. Altogether, these results illustrated that CTCF is a transcription promoter of circSPARC in CRC.

### CircSPARC accelerates CRC progression through the JAK2/STAT3 signalling pathway

**Fig. 4:**
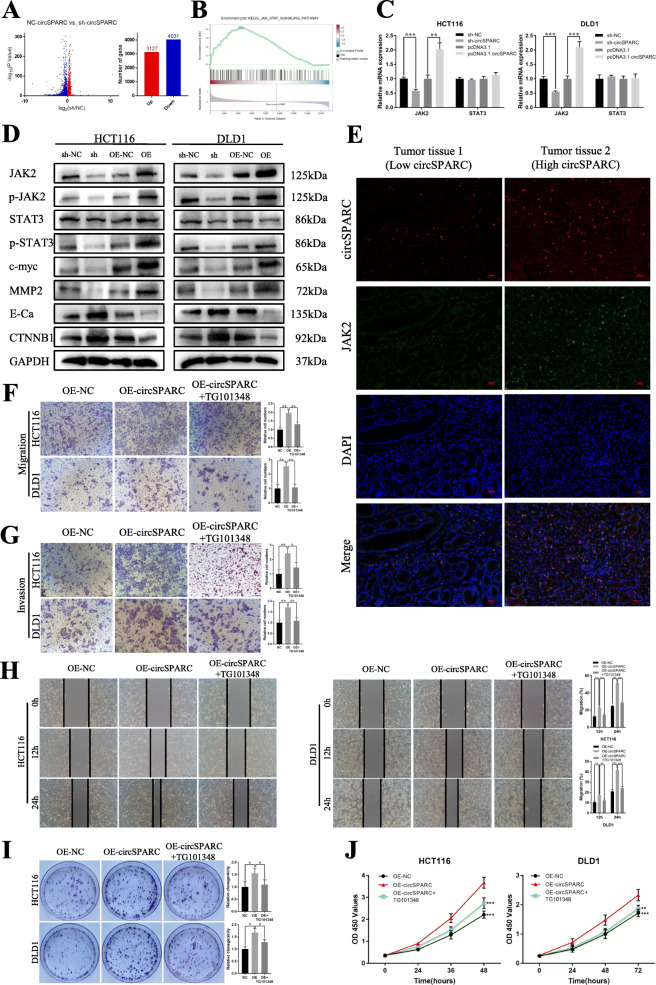
circSPARC regulated the JAK2/STAT3 signalling pathway. **A** and **B.** RNA-seq results showed that circSPARC had connection with JAK/STAT signalling pathway. **C.** RT-qPCR results showed that circSPARC had positive regulation on JAK2 but not STAT3. **D.** Western blot analysis showed that both silencing and overexpression circSPARC can regulate JAK2/STAT3 signalling pathway and its downstream genes expect STAT3. **E.** FISH-IF assay showed that JAK2 expressed higher in CRC tissues with high level of circSPARC. **F** and **G.** The transwell assay showed that the enhanced invasion and migration abilities were reversed by TG101348, an inhibitor of JAK2/STAT3 signalling. **H.** The wound healing assay showed that the enhanced migration abilities were reversed by TG101348, an inhibitor of JAK2/STAT3 signalling. **I** and **J.** The colony formation and CCK-8 assay showed that the enhanced proliferation abilities were reversed by TG101348. *P < 0.05, **P < 0.01, ***P < 0.001 and ****P < 0.0001

To further explore the mechanism of action of circSPARC in CRC, we performed RNA-seq analysis from three pairs of HCT116 cells transfected by lentiviral (sh-NC and sh-circSPARC). We found that when circSPARC was downregulated, 3127 genes were upregulated and 4031 genes were downregulated (Fig. 4A). Among all the genes that were regulated by circSPARC, multiple genes were enriched in the JAK/STAT signalling pathway (Fig. 4B, Additional file Fig. S2E). Since the JAK/STAT3 signalling pathway plays an important role in CRC [17], and the levels of JAK2 and its downstream related mRNAs changed according to our results, we speculated that circSPARC may function through the JAK2/STAT3 signalling pathway. To further confirm this hypothesis, qPCR and western blotting were performed. At the mRNA level, when circSPARC was silenced or overexpressed, JAK2 expression was changed, while the level of STAT3 seemed stable (Fig. 4C). In addition, at the protein level, the expression of JAK2 changed significantly, and its related downstream genes, such as p-JAK2, p-STAT3, E-Cadherin (E-Ca), β-catenin (CTNNB1), MMP2 and c-myc, changed correspondingly, while STAT3 expression was still unchanged (Fig. 4D, Additional file Fig. S2F). Furthermore, FISH-IF staining assay results showed that JAK2 were highly expressed in the tissue with high level of circSPARC (Fig. 4E).

To clarify whether the JAK2/STAT3 pathway mediates the regulatory effect of circSPARC on CRC progression, we used TG101348, an inhibitor of JAK2/STAT3 signalling (especially JAK2), to conduct rescue assays in circSPARC-overexpressing cells. As a result, the stimulative effect of circSPARC overexpression on the metastasis and proliferation ability of CRC cells was clearly reversed in the presence of TG101348 (Fig. 4F-J). Overall, JAK/STAT3 signalling mediates the promoting effect of circSPARC on CRC development. Together, these results indicated that circSPARC promotes the autophosphorylation of JAK2 to p-JAK2 by increasing the expression of JAK2 mRNA, resulting in an increase in p-STAT3 without changing STAT3 expression.

### CircSPARC sponges miR-485-3p in CRC cells

**Fig. 5:**
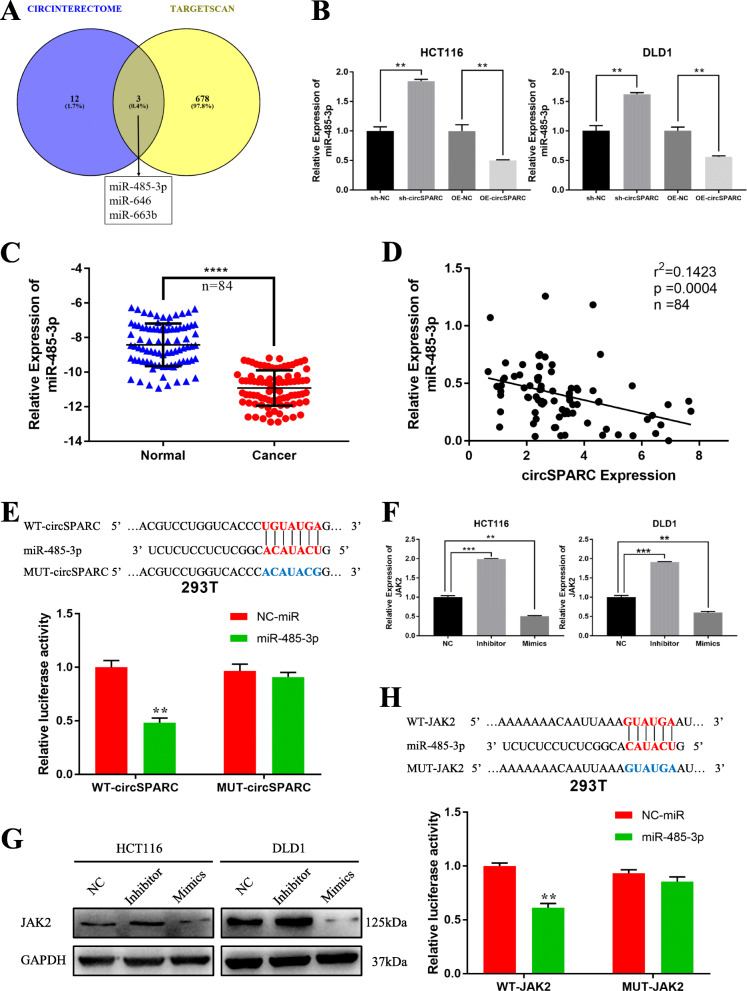
miR-485-3p was directly bound to circSPARC and suppresses JAK2 activity. **A** and **B.** miR-485-3p, miR-646 and miR-663b were predicted that they can bind to circSPARC by two databases and were verified in CRC cells. **C.** RT-qPCR was utilized to analyse the miR-485-3p expression in 84 pairs of CRC tissues and corresponding adjacent non-tumoral tissues. **D.** The negative interaction within miR-485-3p and circSPARC. **E.** Luciferase reporter assay functionally verified the interaction within the circSPARC and miR-485-3p in 293 T cell. **F** and **G.** RT-PCR and western blot analysis detected the JAK2 level with transfecting miR-485-3p mimics or inhibitor in CRC cell lines. **H.** Luciferase reporter assay functionally verified the interaction within the JAK2 and miR-485-3p in 293 T cell. *P < 0.05, **P < 0.01, ***P < 0.001 and ****P < 0.0001

Since circRNAs predominantly located in the cytoplasm usually function as miRNA sponges [18], we further explored whether circSPARC could interact with one or more miRNAs to regulate JAK2 mRNA. We searched CircInteractome (https://circinteractome.nia.nih.gov/) to predict the miRNAs that can interact with circSPARC and used TargetScan (https://www.targetscan.org/vert_72/) to explore the miRNAs that can bind to JAK2. Three potential target miRNAs (miR-485-3p, miR-646 and miR-663b) were predicted to interact with both circSPARC and JAK2 (Fig. 5A), and these miRNAs were selected as candidate miRNAs for subsequent experiments. Among the three candidate miRNAs, only miR-485-3p was increased with circSPARC knockdown, while its level was decreased with circSPARC overexpression in both HCT116 and DLD1 cells (Fig. 5B, Additional file Fig. S3A). Analysis of the 84 pairs of tissues described above indicated that miR-485-3p expression was lower in tumours and there was a negative correlation between circSPARC and miR-485-3p expression (Fig. 5C, D). Meanwhile, the effects of mimics and inhibitor of miR-485-3p were verified (Additional file Fig. S3B). To further confirm the targeted relationship between circSPARC and miR-485-3p, we performed a luciferase reporter assay. The sequence of circSPARC with wild-type or mutant miR-485-3p binding sites was inserted into the psiCHECK-2 vector to perform luciferase reporter assays. After HEK293T cells were co-transfected with miRNA mimics and psiCHECK-2 vectors for 48 h, Rluc activity was detected. Upregulation of miR-485-3p significantly decreased relative Rluc activity, suggesting that miR-485-3p could interact with circSPARC (Fig. 5E). Overall, these results suggested that circSPARC might function as a sponge of miR-485-3p.

### MiR-485-3p targets JAK2 in CRC cells

To identify the relationship between miR-485-3p and JAK2, RT-qPCR, western blot and luciferase reporter assays were performed. We found that miR-485-3p overexpression significantly reduced JAK2 mRNA and protein levels in CRC cells. Conversely, downregulating miR-485-3p showed the opposite result (Fig. 5F, G). The luciferase reporter assay showed that compared to miR-NC, miR-485-3p overexpression significantly reduced the activity of the luciferase reporter containing the wild-type JAK2 sequence (WT); however, this effect disappeared when the miR-485-3p binding sites were mutated (Mut) (Fig. 5H). Collectively, these results indicated that miR-485-3p promoted the invasion and proliferation of CRC cells by targeting JAK2.

### CircSPARC upregulates JAK2 expression by sponging miR-485-3p

**Fig. 6:**
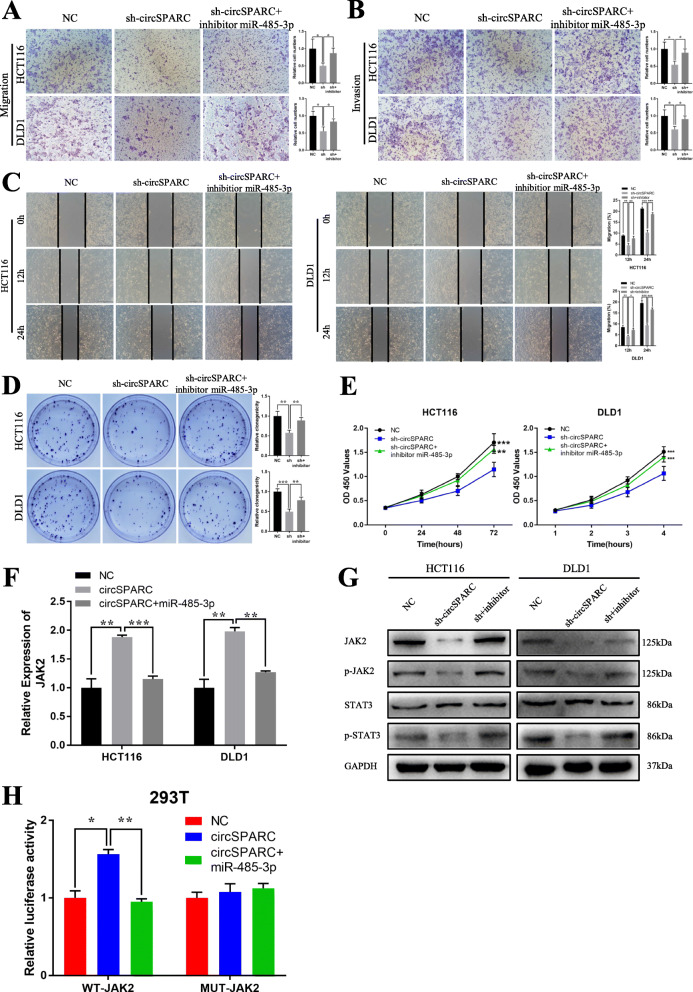
circSPARC regulates JAK2 expression and inhibits CRC cells activities by targeting miR-485-3p. **A** and **B.** The transwell assay demonstrated that cell migration and invasion abilities of CRC cells transfected with sh-circSPARC were counteracted when co-transfected with miR-485-3p inhibitor. **C.** The wound healing assay demonstrated that cell migration abilities of CRC cells transfected with sh-circSPARC were counteracted when co-transfected with miR-485-3p inhibitor. **D** and **E.** The colony formation and CCK-8 assay demonstrated that cell proliferation abilities of CRC cells transfected with sh-circSPARC were counteracted when co-transfected with miR-485-3p inhibitor. **F.** RT-qPCR results showed that the level of JAK2 transfected with circSPARC plasmid was reversed when co-transfected with miR-485-3p mimics in CRC cells. **G.** Western blot analysis showed that the level of JAK2 and its downstream genes transfected with sh-circSPARC was rescued when co-transfected with miR-485-3p inhibitor in CRC cells. **H.** Luciferase reporter assay functionally showed that overexpression of circSPARC increased the activity while co-transfection of the miR-485-3p mimic could eliminate this effect on the wild-type JAK2 sequence in 293 T cell. *P < 0.05, **P < 0.01, ***P < 0.001 and ****P < 0.0001

To further explore the interaction among circSPARC, miR-485-3p and JAK2, several rescue experiments were conducted. The results of the malignant behaviour of circSPARC and miR-485-3p in CRC cell invasion, migration and proliferation indicated that silencing circSPARC could inhibit the invasion, migration and proliferation of CRC cells. However, co-transfection of sh-circSPARC and the inhibitor of miR-485-3p may counteract this effect (Fig. 6A-E). These experimental results suggested that circSPARC enhances the invasion, migration and proliferation of CRC cells by sponging miR-485-3p to regulate JAK2.

Moreover, the data showed that overexpression of circSPARC significantly upregulated JAK2 mRNA levels, whereas co-transfection of the circSPARC overexpression vector and miR-485-3p mimic may cancel out this effect in HCT116 and DLD1 cells and vice versa (Fig. 6F, Additional file Fig. S3C). Western blot analysis showed that low levels of circSPARC could repress JAK2, p-JAK2, and p-STAT3 expression, while co-transfection of sh-circSPARC and the inhibitor of miR-485-3p reversed this effect, and vice versa (Fig. 6G, Additional file Fig. S3D). In addition, luciferase reporter assays showed that the overexpression of circSPARC could significantly increase the activity of the luciferase reporter; however, co-transfection of the circSPARC overexpression vector and miR-485-3p mimic could eliminate this effect on the wild-type JAK2 sequence (WT), while the effect disappeared when the miR-485-3p binding sites were mutated (Mut), and vice versa (Fig. 6H, Additional file Fig. S3E). These results demonstrated that circSPARC could regulate JAK2 expression by acting as a ceRNA to sponge miR-485-3p.

### CircSPARC facilitates the translocation of STAT3 into the nuclei of CRC cells by recruiting FUS

Recently, a report discovered that the binding of FUS to STAT3 contributes to STAT3 nuclear translocation and activation in CRC [19]. Moreover, the online database CircInteractome (https://circinteractome.nia.nih.gov/api/v2/showuptags?circular_rna_query=hsa_circ_0004104&rbp_query=FUS) indicated a potential interaction between circSPARC and FUS. To further explore whether circSPARC can regulate the JAK2/STAT3 signalling pathway by another mechanism, several experiments were performed.

**Fig. 7:**
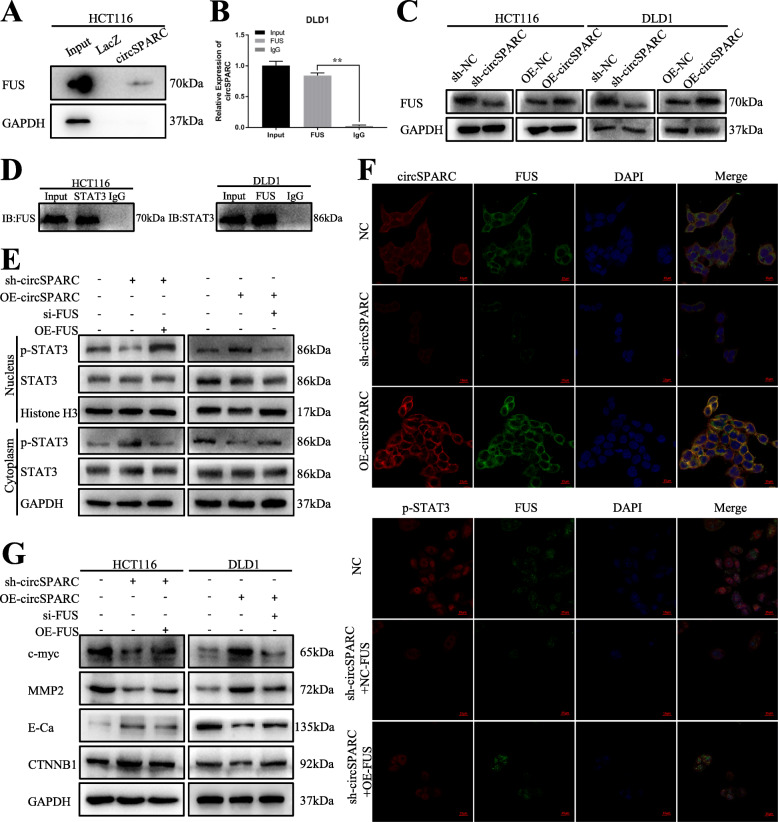
circSPARC intensified the nuclear translocation of STAT3 in CRC via recruiting FUS. **A** and **B.** ChIRP and RIP assay were conducted to prove the interaction between circSPARC and FUS in HCT116 and DLD1 cells. **C.** Western blot assay verified the effect of circSPARC on FUS protein in CRC cells. **D.** The interaction between FUS and STAT3 was confirmed by coIP assay. **E.** Western blot assay was applied to detect the level of STAT3 after nuclear-cytoplasm separation in DLD1 cell. **F.** The co-localization of circSPARC and FUS, as well as that of FUS and STAT3, was validated by IF analysis. *P < 0.05, **P < 0.01, ***P < 0.001 and ****P < 0.0001

The interaction between circSPARC and FUS was verified by ChIRP and RIP assays (Fig. 7A, B). Additionally, the results showed that FUS was positively regulated by circSPARC in CRC cells because its protein level decreased under circSPARC inhibition but increased upon circSPARC stimulation (Fig. 7C). Moreover, the interaction of FUS with STAT3 in CRC cells was proven by a co-IP assay (Fig. 7D). Finally, we confirmed that FUS knockdown reversed the upregulation of nuclear p-STAT3 in circSPARC-overexpressing CRC cells, with the level of total STAT3 unchanged in DLD1 cells (Fig. 7E). Furthermore, in HCT116 cells, IF staining indicated that knockdown of circSPARC expression decreased the nuclear translocation of p-STAT3, whereas this decrease was reversed in the context of simultaneous FUS overexpression (Fig. 7F). In summary, we verified that circSPARC can facilitate the nuclear translocation of STAT3 to enhance the expression of p-STAT3 and regulate downstream genes by recruiting FUS.

### CircSPARC enhances tumour growth and metastasis in vivo

**Fig. 8:**
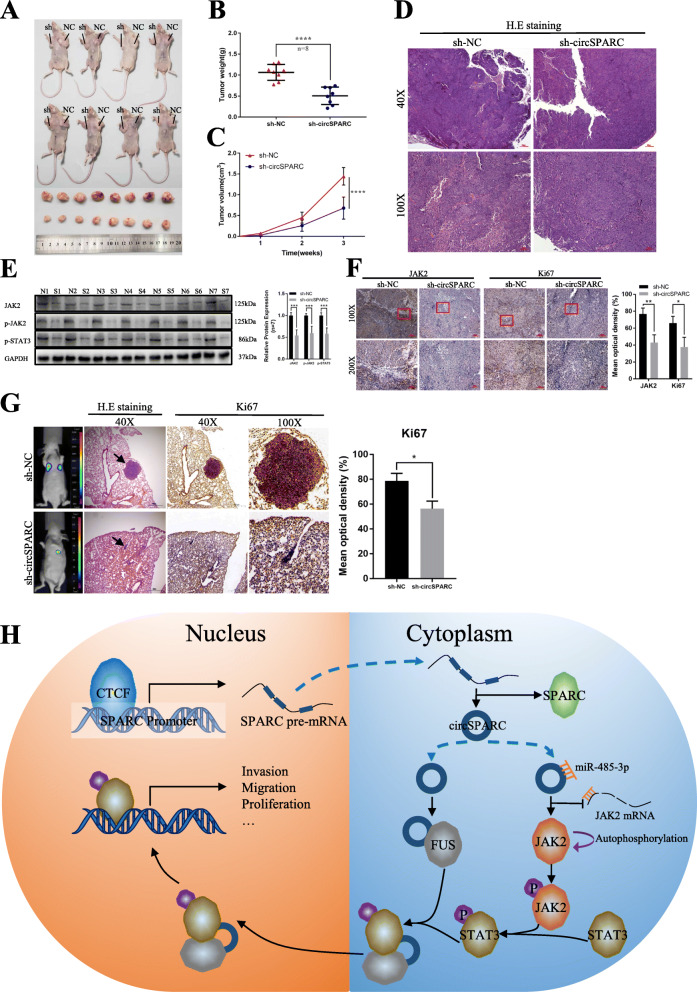
CircSPARC enhances tumour growth and metastasis in vivo. **A-C.** HCT116 cells stably transfected with sh-circSPARC or vector were injected subcutaneously into the BALB/c nude mice. Tumour volume and weight were dramatically decreased in sh-circSPARC group. **D. H** & **E** staining was applied to identify the subcutaneous tumours. **E.** The expression of JAK2 in tumours were analysed by western blot assay. N: sh-NC group. S: sh-circSPARC group. **F.** IHC assay demonstrated the level of JAK2 and Ki67 in pairs of tumours. **G.** After HCT116 cells with knockdown circSPARC injected into the tail vein of nude mice, in vivo fluorescence imaging, the gross lesion in lung tissues, H. E staining and the IHC of Ki67 of metastatic nodules in the lungs were observed. **H.** The hypothetical model depicts the roles of circSPARC in the promotion of CRC. *P < 0.05, **P < 0.01, ***P < 0.001 and ****P < 0.0001

To explore the association between circSPARC and the growth and metastasis of CRC in vivo, HCT116 cells stably transfected with sh-circSPARC or sh-NC vector were subcutaneously injected into BALB/c nude mice. The sizes and weights of the tumours from the sh-circSPARC group were significantly smaller than those of the sh-NC group (Fig. 8A-C). These subcutaneous tumours were further assayed by western blotting, immunohistochemical staining and H&E. The expression of JAK2 and p-STAT3 was significantly downregulated in the sh-circSPARC group compared to the sh-NC group (Fig. 8D-F).

Furthermore, to determine whether circSPARC affected tumour metastasis in vivo, HCT116 cells stably transfected with the sh-NC vector or sh-circSPARC vector were injected into the caudal veins of nude mice. The results demonstrated that there were fewer metastatic foci in the lungs of nude mice in the circSPARC-knockdown groups than in the empty vector group, and micrometastases were detected by H&E (Fig. 8G). Collectively, these results revealed that circSPARC is an oncogene that is associated with proliferation, migration and invasion.

## Discussion

Recently, emerging evidence has suggested that circRNAs play a vital role in cellular development and human diseases, especially cancer [14]. Dysregulated expression of circRNAs might lead to progressive, uncontrolled growth and metastasis of tumours [20]. Using a combination of genomic, biochemical, and cell biological analyses, we proved that circSPARC, a 553 bp exonic circRNA (EcircRNA) containing four exons from its parental gene SPARC, is an oncogenic circRNA in CRC. RNA-seq and clinical specimen analysis demonstrated that circSPARC was upregulated in CRC tissue and induced cell proliferation and metastasis. Moreover, this change in circSPARC levels was correlated with advanced TNM stage, including lymph node metastasis, peripheral metastasis, pathological staging, tumour development and tumorigenesis. Notably, the expression of circSPARC was significantly different in the plasma of normal and cancer patients as well as CRC patients before and after surgery. Currently, the sensitivity of clinical biomarkers for CRC is poor, suggesting that they have very limited application in diagnosis [21]. Therefore, there is a clinical need for an improved tumor biomarker for the diagnosis of CRC. Compare to some other researches of circRNAs, hsa_circ_0002320 has higher sensitivity and specificity for the diagnosis of CRC [22–24]. With the development of liquid biopsy tools and other clinical techniques [25, 26], circSPARC is expected to be a new biomarker for the prognosis and early diagnosis of CRC. All these findings indicated that circSPARC might play a critical role in CRC progression.

Although there have been many reports on the circRNAs overexpressing in CRC, only few studies reported on the reasons for the overexpression. In upstream, we validated that circSPARC in CRC was transcriptionally enhanced by CTCF, a transcription factor to control gene expression in human diseases [27]. A previous study also proved that CTCF was upregulated in CRC and functioned as an oncogene [16]. These findings suggested that upregulation of circSPARC was partially attributed to the high expression of CTCF in CRC.

The JAK/STAT signalling pathway is a classic oncogenic signalling pathway that induces cancer cell migration, growth and differentiation [13]. After the transmission of signals from cytokine family receptors, including the receptors for growth hormone (GH), leptin, and erythropoietin, JAKs undergo dimerization and autophosphorylation to form p-JAKs, which activate STATs into p-STATs, and then the activated p-STATs enhance the transcription of downstream oncogenes [28]. It has been reported that the JAK/STAT signalling pathway, especially JAK2 and STAT3, enhance cell invasion and migration by regulating EMT-related genes, such as repressing E-Cadherin (E-Ca) and β-catenin (CTNNB1) and upregulating MMP2. In addition, this pathway can stimulate cell proliferation by increasing the level of c-myc. As a classical carcinogenic signalling pathway, the JAK2/STAT3 pathway plays an important role in accelerating the progression of CRC and is often associated with poor prognosis in CRC patients. In this study, miR-485-3p, as a target of circSPARC, acted as a ceRNA to regulate the downstream mRNA JAK2. Furthermore, the qPCR and western blot results demonstrated that changes in the expression of circSPARC had a positive effect on JAK2, p-JAK2 and p-STAT3, but STAT3 was unchanged at both the mRNA and protein levels. This result indicated that circSPARC facilitated the activation of p-STAT3 by regulating JAK2 but not STAT3. In summary, circSPARC can increase the level of p-STAT3 via its function as a ceRNA by sponging miR-485-3p to regulate the expression of JAK2.

Currently, reports on the role of circRNAs in promoting nuclear translocation of related proteins are very rare. Few studies reported that some lncRNAs have the ability to help proteins nuclear translocation via combining with RBP. For example, lncRNA-AC020978 could promote the nuclear translocation of PKM2 and regulate PKM2-enhanced HIF-1α transcription activity [29]. In this study, FUS also participated in the regulatory relationship between circSPARC and the JAK2/STAT3 signalling pathway. FUS is a transcription factor and an RBP associated with several kinds of malignant tumours, and it was reported that the binding of FUS to AKT contributes to AKT nuclear translocation and activation [30]. Moreover, FUS can also contribute to STAT3 nuclear translocation and activation in CRC [19]. In this research, RIP and ChIRP assays demonstrated that circSPARC and FUS can interact directly. The results showed that circSPARC acted through RBP and recruited FUS, causing the accumulation of FUS in cells. In summary, circSPARC enhanced the nuclear translocation of p-STAT3 by recruiting FUS. The hypothetical model depiction of circSPARC is shown in Fig. 8H. However, the RNA network has a multilayered, multidirectional, multi-interactive and multidimensional network architecture that orchestrates cellular responses. Whether circSPARC communicates with other miRNAs/lncRNAs/mRNAs/RBPs to form RNA regulatory networks needs to be further confirmed.

## Conclusion

In conclusion, we found that circSPARC functioned as a carcinogenic circRNA during CRC progression and revealed a novel regulatory pathway in which circSPARC upregulated JAK2 expression by sponging miR-485-3p to enhance STAT3 activation. Moreover, circSPARC recruited FUS and facilitated the nuclear translocation of activated STAT3. These findings clearly indicate that circSPARC can regulate the level of p-STAT3 via two different pathways. We identified circSPARC as a cancer-associated circRNA that could be a novel potential diagnostic and therapeutic target for CRC.

## Supplementary Information

**Additional file 1: Figure S1. A.** RT-qPCR was applied to detect the expression of circSPARC in colorectal adenoma tissues and the adjacent normal tissues. The results showed that circSPARC is slightly upregulated in adenoma tissues. **B.** The effect of circSPARC siRNA and change of parental gene SPARC in CRC cells was analysed by RT-qPCR. **C.** The effect of circSPARC plasmid and change of parental gene SPARC in CRC cells was analysed by RT-qPCR and western blot. **D.** The effect of lentiviral and change of parental gene SPARC in CRC cells was analysed by RT-qPCR and western blot. **E.** The activity of CRC cells which were cultured by serum-free medium were detected by CCK-8 assay. The results showed that there was no difference in cell proliferation rate between the treatment group and the control group within 24 h. ns: not significant, *P < 0.05, **P < 0.01, ***P < 0.001 and ****P < 0.0001

**Additional file 2: Figure S2. A.** GEPIA database exhibited that SPARC is upregulated in CRC. B. Starbase database showed that CTCF has a positive correlation with SPARC in CRC. **C.** RT-qPCR indicated the circSPARC expressed higher in CRC cell lines (HCT116 and DLD1) than in the normal colorectal epithelium cell line (FHC). **D.** The effect of CTCF siRNA in CRC cells was analysed by RT-qPCR. **E.** The bubble diagram of RNA-seq analyzed by KEGG pathway showed that circSPARC may have correlation with JAK/STAT signal pathway. **F.** The quantitative analysis of related proteins expression in Fig. 4D. The result showed that both silencing and overexpression circSPARC can regulate JAK2/STAT3 signalling pathway and its downstream genes expect STAT3. *P < 0.05, **P < 0.01, ***P < 0.001 and ****P < 0.0001

**Additional file 3: Figure S3. A.** RT-qPCR was applied to detect the level of miR-646 and miR-663b after silencing or overexpressing circSPARC. **B.** RT-qPCR was applied to detect the effect of miR-485-3p inhibitor and mimics in CRC cells. **C.** RT-qPCR results showed that the level of JAK2 transfected with sh-circSPARC was reversed when co-transfected with miR-485-3p inhibitor in CRC cells. **D.** Western blot analysis showed that the level of JAK2 and its downstream genes transfected with circSPARC plasmid was rescued when co-transfected with miR-485-3p mimics in CRC cells. **E.** Luciferase reporter assay functionally showed that knockdown of circSPARC decreased the activity while co-transfection of the miR-485-3p inhibitor could eliminate this effect on the wild-type JAK2 sequence in 293 T cell. *P < 0.05, **P < 0.01, ***P < 0.001 and ****P < 0.0001

**Additional file 4: Table S1.** Primer names and sequences

**Additional file 5: Table S2.** Antibodies list

## Data Availability

All data that support the findings of this study are available from the corresponding authors upon reasonable request.

## Abbreviations

CRC: Colorectal cancer
circRNA: Circular RNA
miRNA: microRNA
siRNAs: Short interfering RNAs
CCK-8: Cell Counting Kit-8
FISH: Fluorescence in situ hybridization
RT-qPCR: Quantitative real-time PCR
RIP: RNA immunoprecipitation
H&E: Hematoxylin and eosin
IHC: Immunohistochemical staining
RBP: RNA-binding protein
