# Supplementary figures and images for: The circular RNA circSPARC enhances the migration and proliferation of colorectal cancer by regulating the JAK/STAT pathway
Source: Mol Cancer. 2021 Jun 1;20:81. doi: 10.1186/s12943-021-01375-x (PMC8167978; doi:10.1186/s12943-021-01375-x)

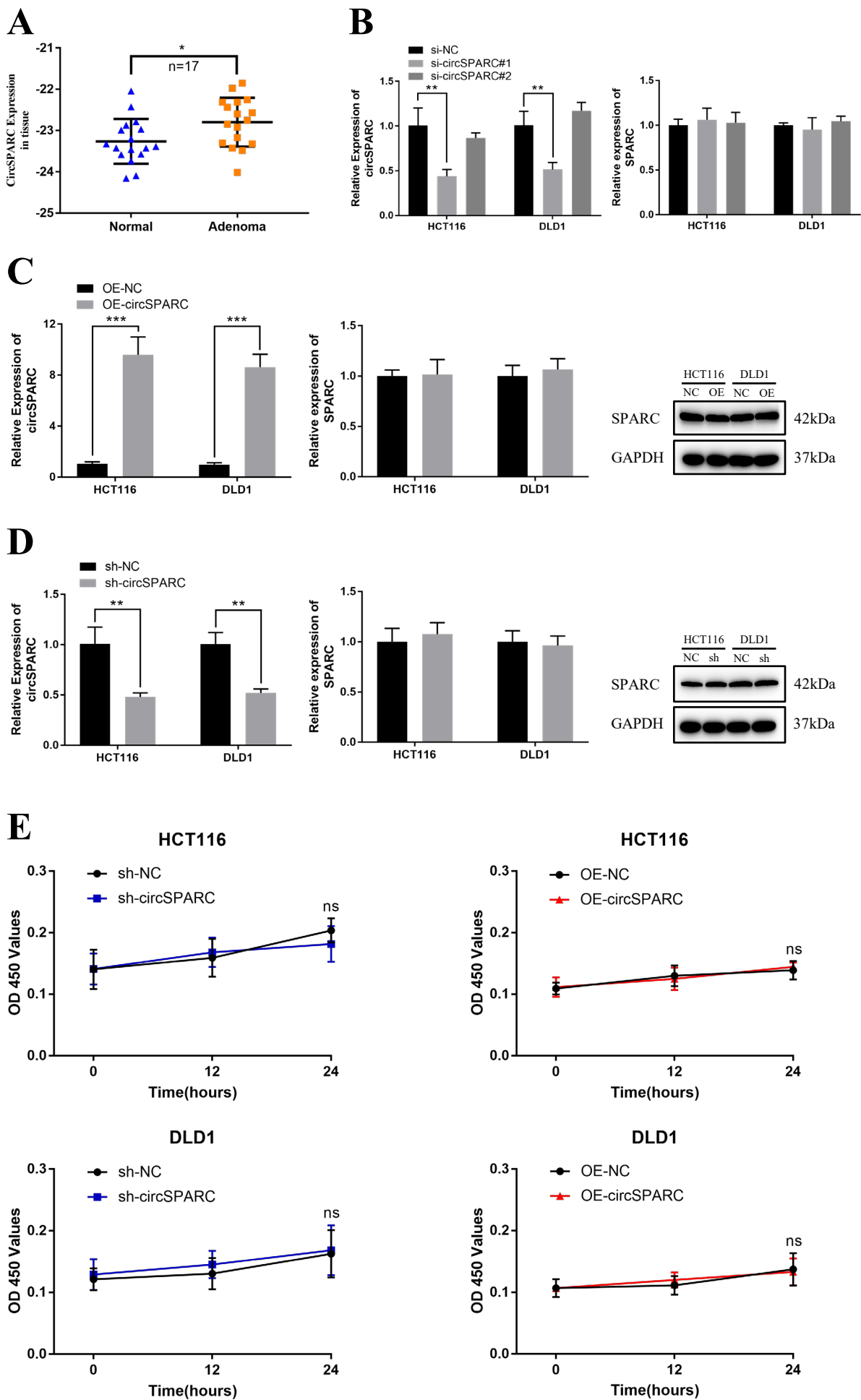

Supplement: Supplementary file 1 — Additional file 1: Figure S1. A. RT-qPCR was applied to detect the expression of circSPARC in colorectal adenoma tissues and the adjacent normal tissues. The results showed that circSPARC is slightly upregulated in adenoma tissues. B. The effect of circSPARC siRNA and change of parental gene SPARC in CRC cells was analysed by RT-qPCR. C. The effect of circSPARC plasmid and change of parental gene SPARC in CRC cells was analysed by RT-qPCR and western blot. D. The effect of lentiviral and change of parental gene SPARC in CRC cells was analysed by RT-qPCR and western blot. E. The activity of CRC cells which were cultured by serum-free medium were detected by CCK-8 assay. The results showed that there was no difference in cell proliferation rate between the treatment group and the control group within 24 h. ns: not significant, *P < 0.05, **P < 0.01, ***P < 0.001 and ****P < 0.0001 [file 12943_2021_1375_MOESM1_ESM.pdf]

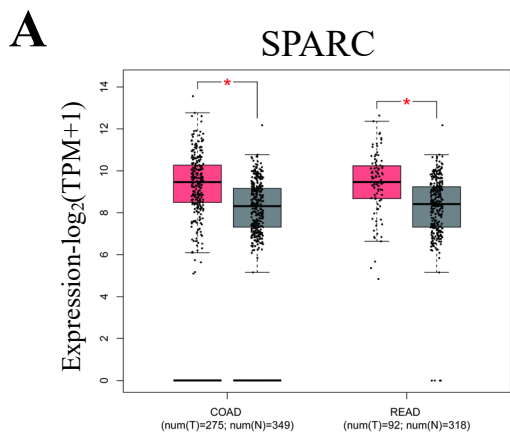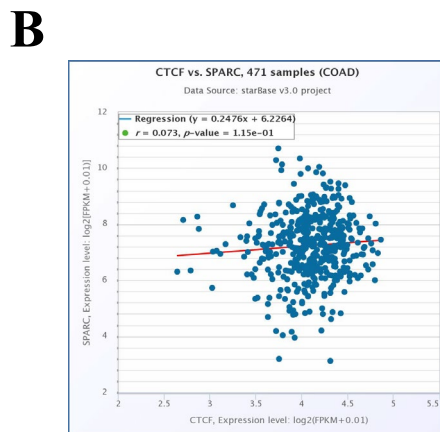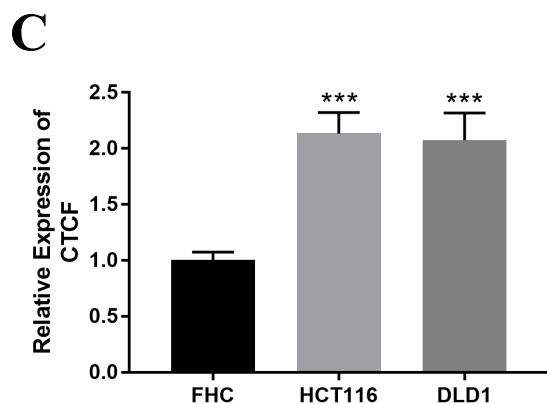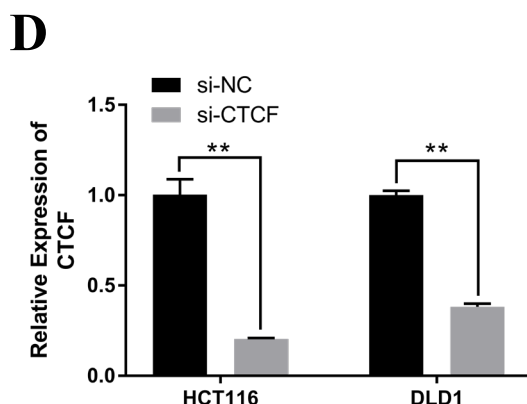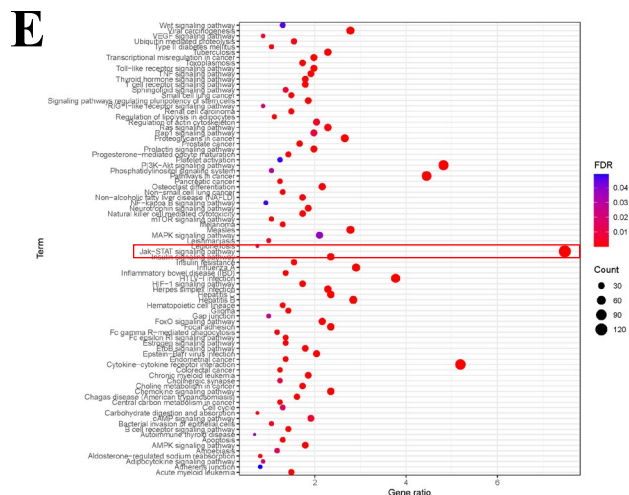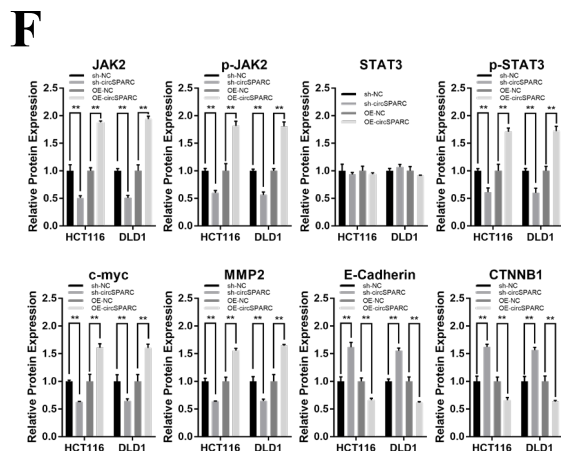

Supplement: Supplementary file 2 — Additional file 2: Figure S2. A. GEPIA database exhibited that SPARC is upregulated in CRC. B. Starbase database showed that CTCF has a positive correlation with SPARC in CRC. C. RT-qPCR indicated the circSPARC expressed higher in CRC cell lines (HCT116 and DLD1) than in the normal colorectal epithelium cell line (FHC). D. The effect of CTCF siRNA in CRC cells was analysed by RT-qPCR. E. The bubble diagram of RNA-seq analyzed by KEGG pathway showed that circSPARC may have correlation with JAK/STAT signal pathway. F. The quantitative analysis of related proteins expression in Fig. 4D. The result showed that both silencing and overexpression circSPARC can regulate JAK2/STAT3 signalling pathway and its downstream genes expect STAT3. *P < 0.05, **P < 0.01, ***P < 0.001 and ****P < 0.0001 [file 12943_2021_1375_MOESM2_ESM.pdf]

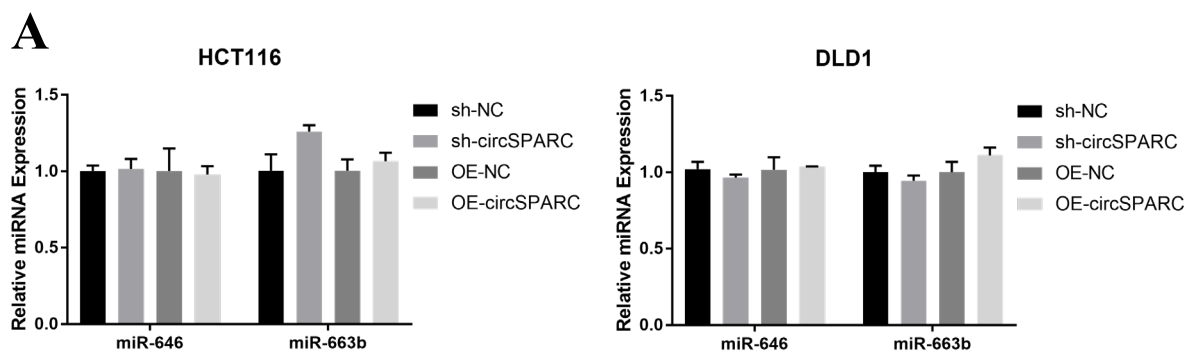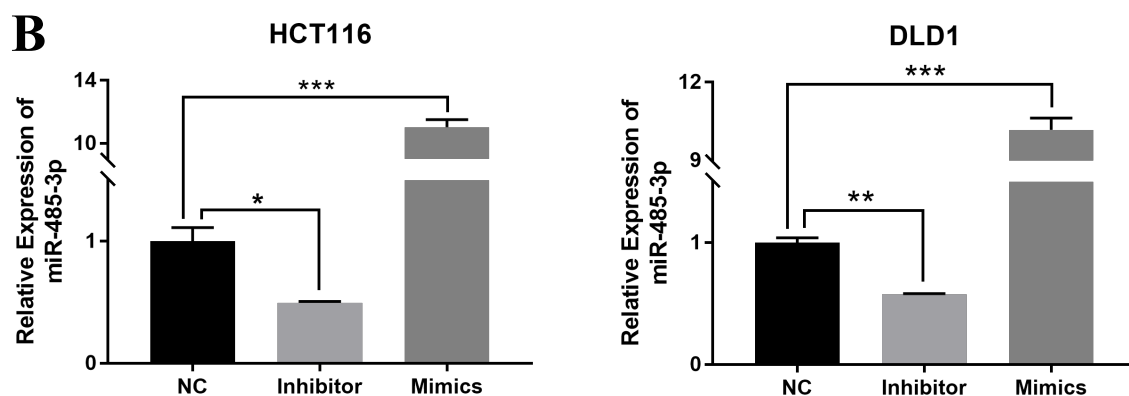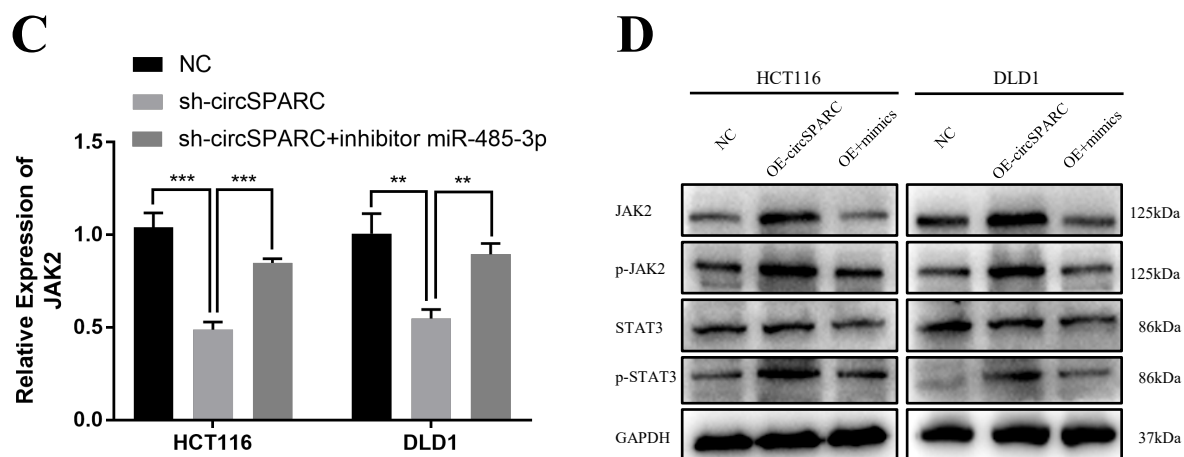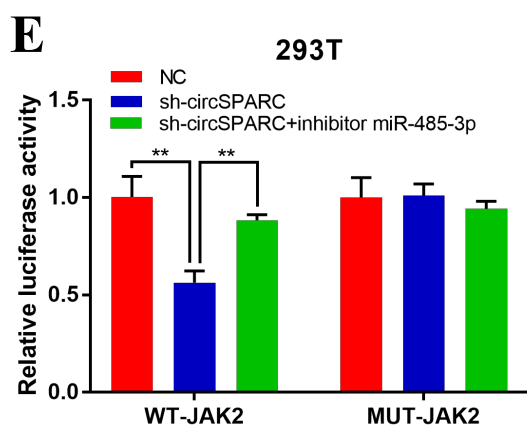

Supplement: Supplementary file 3 — Additional file 3: Figure S3. A. RT-qPCR was applied to detect the level of miR-646 and miR-663b after silencing or overexpressing circSPARC. B. RT-qPCR was applied to detect the effect of miR-485-3p inhibitor and mimics in CRC cells. C. RT-qPCR results showed that the level of JAK2 transfected with sh-circSPARC was reversed when co-transfected with miR-485-3p inhibitor in CRC cells. D. Western blot analysis showed that the level of JAK2 and its downstream genes transfected with circSPARC plasmid was rescued when co-transfected with miR-485-3p mimics in CRC cells. E. Luciferase reporter assay functionally showed that knockdown of circSPARC decreased the activity while co-transfection of the miR-485-3p inhibitor could eliminate this effect on the wild-type JAK2 sequence in 293 T cell. *P < 0.05, **P < 0.01, ***P < 0.001 and ****P < 0.0001 [file 12943_2021_1375_MOESM3_ESM.pdf]
