# Supplementary material for: The circular RNA circSPARC enhances the migration and proliferation of colorectal cancer by regulating the JAK/STAT pathway
Source: Mol Cancer. 2021 Jun 1;20:81. doi: 10.1186/s12943-021-01375-x (PMC8167978; doi:10.1186/s12943-021-01375-x)
Supplement: Supplementary file 4 — Additional file 4: Table S1. Primer names and sequences [file 12943_2021_1375_MOESM4_ESM.docx]

**Table S1. Primer names and sequences**

| **Primer names** | **Primer sequences** |
| --- | --- |
| circSPARC  (hsa_circ_0004104) | Forward: ACATCGCCCTGGATGAGTGG |
|  | Reverse: AGCTTGTGGCCCTTCTTGGT |
| hsa_circ_0081069 | Forward: AGTTGGACTTGGCCCTGGAC |
|  | Reverse: AGCACCTGCAGGACCAGTTT |
| hsa_circ_0044556 | Forward: CGCAGATGGTGTTGCTGGTC |
|  | Reverse: CAGTCAGACCCTTGGCACCA |
| hsa_circ_0057362 | Forward: TGAAAGGACACAGAGGCTTCGA |
|  | Reverse: CATGGGTCCAGGAGCTCCAT |
| hsa_circ_0057360 | Forward: CGACCTGGAGAGCGAGGATT |
|  | Reverse: AGGAGCACCTGTTTCACCCT |
| hsa_circ_0058123 | Forward: AGAGAGCCCCAAAGCCACTG |
|  | Reverse: GAAGTTGTGGCTGCAGGTCC |
| hsa_circ_0002447 | Forward: AGCTCTTAGCCAGCACTCGC |
|  | Reverse: AGTCCCAGTGCAGATGAGCC |
| hsa_circ_0092283 | Forward: CCCCCTCTGGCTTCTGTGTT |
|  | Reverse: AGGAAGGTGGCAGCAGGATC |
| hsa_circ_0007482 | Forward: ACGAGCAGGGTATCCAGCAG |
|  | Reverse: AGGTTGATGCCCCGGAAGAG |
| hsa_circ_0090364 | Forward: GGAAGGACCTCATCAGTGCCA |
|  | Reverse: GAACGGTGTCTGGACTGGCT |
| 18S | Forward: GTAACCCGTTGAACCCCATT |
|  | Reverse: CCATCCAATCGGTAGTAGCG |
| miR-485-3p | RT Primer:  GTCGTATCCAGTGCAGGGTCCGAGGTATTCGCACTGGATACGACAGAGAG |
|  | Forward: GCGGTCATACACGGCTCTC |
|  | Reverse: AGTGCAGGGTCCGAGGTATT |
| miR-646 | RT Primer:  GTCGTATCCAGTGCAGGGTCCGAGGTATTCGCACTGGATACGACGCCTCA |
|  | Forward: CGCGAAGCAGCTGCCTC |
|  | Reverse: AGTGCAGGGTCCGAGGTATT |
| miR-663b | RT Primer:  GTCGTATCCAGTGCAGGGTCCGAGGTATTCGCACTGGATACGACCCTCAG |
|  | Forward: GGTGGCCCGGCCGTGC |
|  | Reverse: AGTGCAGGGTCCGAGGTATT |
| U6 | Forward: GCTTCGGCAGCACATATACT |
|  | Reverse: GTGCAGGGTCCGAGGTATTC |
| SPARC | Forward: CAATGACAACAAGACCTTCGAC |
|  | Reverse: GAATTCGGTCAGCTCAGAGTC |
| JAK2 | Forward: CGAATGGTGTTTCTGATGTACC |
|  | Reverse: GTCTCCTACTTCTCTTCGTACG |
| STAT3 | Forward: TCGGCTAGAAAACTGGATAACG |
|  | Reverse: TGCAACTCCTCCAGTTTCTTAA |
| GAPDH | Forward: AAGGTCGGAGTCAACGGATTTG |
|  | Reverse: CCATGGGTGGAATCATATTGGAA |
| SPARC pre-mRNA | Forward: TCTCCTCACTCCCAAACACCA |
|  | Reverse: CCACCTCCCATTACACCCAT |
| P1 | Forward: TGTCTCAAAAAAAAAAAAAAAATCCTGGGG |
|  | Reverse: GAATACCATGGAAAGGAAGAAGGC |
| P2 | Forward: AAACCCAACCCATGCTTCC |
|  | Reverse: AAAAGTACAAGGAATTCAATCTGTGAC |
| P3 | Forward: TTTCCCTTCTCAGTTCTGCACTTAA |
|  | Reverse: TTGTATTTTTAGTAGAAATGGGGTTTCAAC |
| P4 | Forward: TAAATTAGCTGGGTGTTGTGGC |
|  | Reverse: GTGGTTTCCGGTTTTTTTGTTTG |
| P5 | Forward: AAAACTTTTTGAGGACAAGGACCA |
|  | Reverse: CATAGATGCTAAGTAACTTGCCTATGA |
| P6 | Forward: TTTCCTTATTATTAAAATGGTCATAATTACAATGCC |
|  | Reverse: TGTACCACTAATAGACACATAAGGGA |
| P7 | Forward: AGTGGAGTTTGAGTGGGATTT |
|  | Reverse: TCTAACCAGTGAGCTTTACAGGA |
| P8 | Forward: AGATCAAGACACTTGGGCC |
|  | Reverse: ACCAAACGTCCCAACCC |
| P9 | Forward: CAGGACAGCCCACCGCA |
|  | Reverse: GAAGGACCGCGGGAATG |
