# Supplementary material for: The circular RNA circSPARC enhances the migration and proliferation of colorectal cancer by regulating the JAK/STAT pathway
Source: Mol Cancer. 2021 Jun 1;20:81. doi: 10.1186/s12943-021-01375-x (PMC8167978; doi:10.1186/s12943-021-01375-x)
Supplement: Supplementary file 5 — Additional file 5: Table S2. Antibodies list [file 12943_2021_1375_MOESM5_ESM.docx]

**Table S2. Antibodies list**

| **Name** | **Company** | **Number** |
| --- | --- | --- |
| Anti-JAK2 | Cell Signaling Technology | #3230 |
| Anti-p-JAK2 (phospho Y1007+Y1008) | Abcam | ab32101 |
| Anti-STAT3 | Cell Signaling Technology | #9139 |
| Anti-p-STAT3 (phospho Y705) | Abcam | ab76315 |
| Anti-c-myc | Cell Signaling Technology | #18583 |
| Anti-MMP2 | Cell Signaling Technology | #40994 |
| Anti-E-Cadherin | Cell Signaling Technology | #3195 |
| Anti-CTNNB1 | Cell Signaling Technology | #8480 |
| Anti-FUS | Cell Signaling Technology | #67840 |
| Anti-CTCF | Abcam | Ab128873 |
| Anti-SPARC | Cell Signaling Technology | #5420 |
| Anti-GAPDH | Abcam | ab8245 |
| Anti-Histone H3 | Abcam | ab1791 |
| Goat Anti-Rabbit IgG H&L (HRP) | Abcam | ab6721 |
| Goat Anti-Mouse IgG H&L (HRP) | Abcam | ab6789 |
| Goat Anti-Rabbit IgG H&L  (Alexa Fluor® 647) | Abcam | ab150079 |
| Goat Anti-Rabbit IgG H&L  (Alexa Fluor® 488) | Abcam | ab150077 |
| Goat Anti-Mouse IgG H&L  (Alexa Fluor® 488) | Abcam | ab150113 |
